# TLR-3 stimulation improves anti-tumor immunity elicited by dendritic cell exosome-based vaccines in a murine model of melanoma

**DOI:** 10.1038/srep17622

**Published:** 2015-12-03

**Authors:** Martina Damo, David S. Wilson, Eleonora Simeoni, Jeffrey A. Hubbell

**Affiliations:** 1 Institute of Bioengineering, School of Life Sciences and School of Engineering, Ecole Polytechnique Fédérale Lausanne, CH-1015 Lausanne, Switzerland; 2 Center of PhenoGenomics, School of Life Sciences, Ecole Polytechnique Fédérale Lausanne, CH-1015 Lausanne, Switzerland; 3 Institute for Chemical Science and Engineering, School of Basic Sciences, Ecole Polytechnique Fédérale Lausanne, CH-1015 Lausanne, Switzerland; 4 Institute for Molecular Engineering, University of Chicago, IL 60637, USA

## Abstract

Dendritic cell (DC)-derived exosomes (Dexo) contain the machinery necessary to activate potent antigen-specific immune responses. As promising cell-free immunogens, Dexo have been tested in previous clinical trials for cancer vaccine immunotherapy, yet resulted in limited therapeutic benefit. Here, we explore a novel Dexo vaccine formulation composed of Dexo purified from DCs loaded with antigens and matured with either the TLR-3 ligand poly(I:C), the TLR-4 ligand LPS or the TLR-9 ligand CpG-B. When poly(I:C) was used to produce exosomes together with ovalbumin (OVA), the resulting Dexo vaccine strongly stimulated OVA-specific CD8^+^ and CD4^+^ T cells to proliferate and acquire effector functions. When a B16F10 melanoma cell lysate was used to load DCs with tumor antigens during exosome production together with poly(I:C), we obtained a Dexo vaccine capable of inducing robust activation of melanoma-specific CD8^+^ T cells and the recruitment of cytotoxic CD8^+^ T cells, NK and NK-T cells to the tumor site, resulting in significantly reduced tumor growth and enhanced survival as compared to a Dexo vaccine formulation similar to the one previously tested on human patients. Our results indicate that poly(I:C) is a particularly favorable TLR agonist for DC maturation during antigen loading and exosome production for cancer immunotherapy.

Immunotherapy for cancer aims at stimulating tumor-specific immune responses to prevent, treat or eradicate malignancies^1^,^2^. Several approaches have been exploited clinically for cancer immunotherapy, including the use of dendritic cells (DCs) for therapeutic vaccination. As professional antigen-presenting cells (APCs), DCs represent a favorable candidate for immunotherapy purposes due to their ability to take up, process and present antigens and to sense danger signals to initiate an effective cancer-specific immune response. However, DC-based therapies are far from optimal, since *ex vivo* or *in vivo* manipulation of patient-derived DCs is still time-consuming, costly and associated with risks and a high rate of failure^3^,^4^.

In recent years, alternative approaches to the use of DCs in cancer vaccination have been investigated, including the use of exosomes. Exosomes are 30–150 nm membrane vesicles originating from intracellular multivesicular bodies and secreted into the extracellular space by most eukaryotic cell types^5^,^6^. In particular, exosomes originating from DCs (Dexo) contain several immunologically relevant components, such as antigens, MHC class I and II molecules (often complexed with antigenic epitopes), co-stimulatory molecules (e.g., CD80, CD86, CD40), cellular adhesion molecules (e.g., ICAM-1) and integrins^7^,^8^. Since exosomes can transfer their protein and nucleic acid content from a secreting cell to a target cell, Dexo are considered to be important intercellular communication vehicles exploited by DCs in the orchestration of immune responses^7^,^8^,^9^,^10^.

Murine Dexo have been shown to be able to stimulate antigen-specific CD4^+^ and CD8^+^ T cells both *in vitro* and *in vivo* and to enhance anti-cancer immunity *in vivo*^11^,^12^,^13^,^14^,^15^,^16^,^17^. Antigen-loaded Dexo derived from the DCs of cancer patients have been tested in phase I clinical trials for the treatment of melanoma and non-small cell lung carcinoma. Those clinical trials proved the feasibility and safety of Dexo-based vaccination in human cancer patients but did not show significant tumor growth control or regression in the treated candidates^18^,^19^,^20^. One hypothesis is that these antigen-loaded Dexo did not contain the activation signals required to elicit and activate cytotoxic effector cells that would be able to recognize and kill transformed cells.

Maturation of DCs via treatment with toll-like receptor (TLR) ligands as adjuvants to activate danger signal-sensing pathways coupled to antigen loading of DCs instead of exosomes has been proposed as a possible solution to improve the immunogenic profile of Dexo. Recently, it was shown that treatment of DCs with LPS (a TLR-4 ligand) or Pam_3_ (a TLR-1/2 ligand) leads to secretion of Dexo with an increased ability to stimulate cytotoxic natural killer (NK) and CD8^+^ T cells and significantly affect tumor growth *in vivo*^16^,^21^,^22^. However, LPS is not clinically viable and the effect of other adjuvants, already proposed for clinical use in immunotherapies, has yet to be explored in the development of Dexo-based vaccines.

In an attempt to further improve the immune stimulatory properties of Dexo and to provide a vaccination tool easily transferrable to clinical development for both infectious diseases and cancer, we compared poly(I:C) (a TLR-3 ligand) and CpG-B (a TLR-9 ligand) to LPS as adjuvants for DC maturation during Dexo production^23^,^24^,^25^,^26^,^27^,^28^. Our results indicate that Dexo produced upon treatment of DCs with the model antigen ovalbumin (OVA) and poly(I:C) (Dexo(OVA + pIC)) robustly activate OVA-specific Th1 immune responses, characterized by the release of the pro-inflammatory cytokines interferon-γ (IFNγ) and tumor necrosis factor-α (TNFα) by CD4^+^ T lymphocytes and associated with the stimulation of OVA-specific cytotoxic CD8^+^ T cells but negligible production of OVA-specific antibodies. Most importantly, therapeutic vaccination targeted to the tumor-draining lymph nodes (tdLNs) of B16F10 melanoma-bearing mice with Dexo released by DCs co-cultured with oxidized necrotic B16F10 cells as source of melanoma antigens and matured with poly(I:C) (Dexo(B16 + pIC)) raised both melanoma-specific effector CD8^+^ T cells in the tdLNs, spleen and tumor mass and tumor-infiltrating NK and NK-T cells, significantly reducing tumor growth and increasing the survival rate of diseased mice.

## Results

### Characterization of DC-derived exosomes produced in the presence of LPS, CpG-B or poly(I:C)

DCs express a wide range of pattern-recognition receptors, among which TLR-3, TLR-4 and TLR-9 are good candidates as adjuvant targets to elicit potent cytotoxic T cell responses in mice and humans for cancer as well as infectious disease vaccination^25^,^26^,^27^,^28^,^29^. In an attempt to improve the immunogenic profile of DC-derived exosomes (Dexo) and test their ability to raise antigen-specific immune responses against the model antigen OVA, Dexo were harvested from the supernatant of bone marrow-derived DCs (BMDCs) either left untreated (Dexo(unt)), treated with OVA (Dexo(OVA)) or treated with OVA and matured with either the TLR-4 ligand LPS (Dexo(OVA + LPS)), the synthetic oligonucleotide CpG-B ligand of TLR-9 (Dexo(OVA + CpGB)) or the synthetic dsRNA analog poly(I:C) ligand of TLR-3 (Dexo(OVA + pIC)).

**Figure 1:**
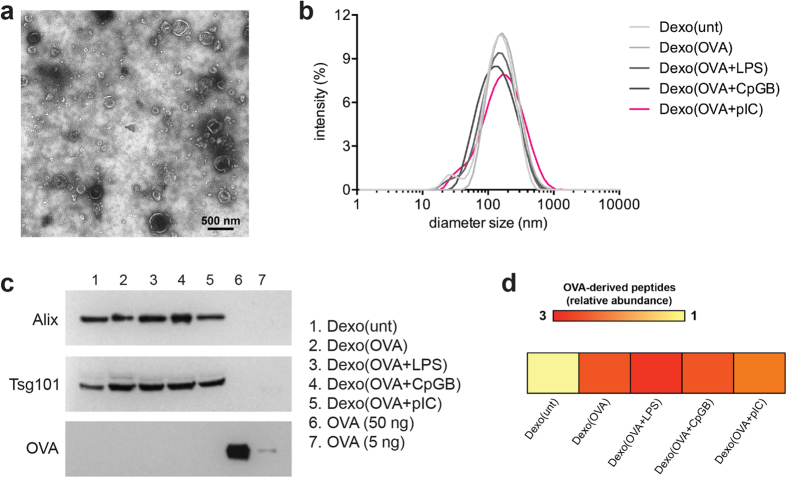
Exosomes are purified from the supernatant of DCs cultured in the presence of the model antigen ovalbumin and activated with different Toll-like receptor ligands. Exosomes were purified from the supernatant of untreated BMDCs (Dexo(unt)) or of BMDCs treated with OVA (Dexo(OVA)) or from the supernatant of BMDCs treated with OVA and matured with LPS (Dexo(OVA + LPS)), CpG-B (Dexo(OVA + CpGB) or poly(I:C) (Dexo(OVA + pIC)). (**a**) Example of transmission electron microscopy of one of the Dexo samples after purification from the supernatant of BMDCs confirms the expected physical characteristics of exosomal vesicles. (**b**) Diameter of exosomes was measured by DLS analysis of the different Dexo samples. (**c**) Presence of the exosome-specific markers Alix (100 kDa) and Tsg101 (46 kDa) and of full-length OVA protein (45 kDa) was detected by western blot to confirm the identity of *bona-fide* exosomes and loading of intact OVA in Dexo samples. 50 ng and 5 ng of OVA were used as positive control for detection of full-length OVA. (**d**) Presence of OVA-derived peptides was detected in Dexo samples by mass spectrometry. Abundance of OVA-related peptides in Dexo(OVA), Dexo(OVA + LPS), Dexo(OVA + CpGB) and Dexo(OVA + pIC) samples is normalized to the background abundance of OVA-related peptides detected in Dexo(unt) samples (relative abundance).

Transmission electron microscopy and dynamic light scattering (DLS) analysis of Dexo(unt), Dexo(OVA), Dexo(OVA + LPS), Dexo(OVA + CpGB) and Dexo(OVA + pIC) samples confirmed the presence of 30–150 nm cup-shaped vesicles, proving that TLR stimulation of exosome-secreting DCs does not affect Dexo physical characteristics (Fig. 1a,b). While typical exosomal markers such as the regulators of vesicular trafficking Alix and Tsg101 were revealed by western blot in all the Dexo preparations, full-length OVA could not be detected, suggesting the presence of only processed OVA peptides (Fig. 1c). In fact, OVA-related peptides, measured by mass spectrometry as albumin peptides, could be detected more abundantly in those Dexo formulations prepared from OVA-treated BMDCs as compared to Dexo(unt) samples (Fig. 1d).

### Screening of the immunogenic profile of Dexo(unt), Dexo(OVA), Dexo(OVA+LPS), Dexo(OVA+CpGB) and Dexo(OVA+pIC) in an OT-I adoptive transfer model of vaccination

**Figure 2:**
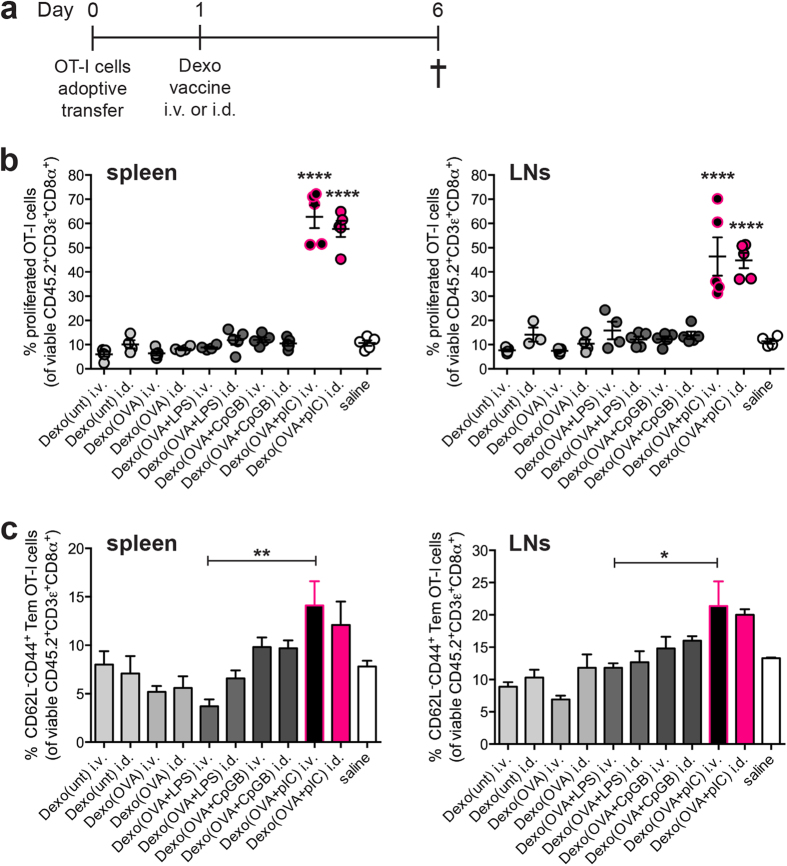
Vaccination with exosomes from OVA-loaded and poly(I:C)-activated DCs strongly activates proliferation and acquisition of effector functions of adoptively transferred OT-I OVA-specific CD8^+^ T cells *in vivo*. (**a**) CFSE-labeled OT-I CD8^+^ T cells (CD45.2^+^) were intravenously transferred into CD45.1^+^ recipient mice on day 0. Recipient mice were vaccinated on day 1 with 50 μg of the indicated Dexo formulation or saline. On day 6, spleens and LNs were collected to analyze OT-I cells. (**b**) Proliferation was measured by flow cytometry as dilution of CFSE dye in adoptively transferred CD45.2^ +^ CD3ε^ +^CD8α^+^ OT-I cells retrieved from the spleen (left) and LNs (right). (**c**) Acquisition of the CD62L^-^CD44^+^ effector memory phenotype of adoptively transferred CD45.2^+^ CD3ε^+^ CD8α^+^ OT-I cells was measured by flow cytometric analysis of cells harvested from the spleen (left) and LNs (right). Data represent mean ± SEM from 2 independent experiments (N = 10). Statistical analysis was performed by one-way ANOVA and Bonferroni *post-hoc* test correction. In (**b**) statistics represent comparisons between Dexo(OVA + pIC) i.v. with both Dexo(OVA + LPS) i.v. and Dexo(OVA + CpGB) i.v. groups or between Dexo(OVA + pIC) i.d. with both Dexo(OVA + LPS) i.d. and Dexo(OVA + CpGB) i.d. groups. In (**c**) statistics represent comparisons between the indicated experimental groups. ^*^P < 0.05 ^**^P < 0.01 and ^****^P < 0.0001.

Antigen-specific cytotoxic CD8^+^ T cells play a major role in anti-cancer immunity by directly recognizing and killing transformed cells. To compare the ability of the Dexo formulations produced from BMDCs incubated in the presence of LPS, CpG-B or poly(I:C) to activate an antigen-specific cellular immune response *in vivo*, we took advantage of an OT-I adoptive transfer model of vaccination (Fig. 2a). Recipient mice were vaccinated either intravenously (i.v.) or intradermally (i.d.) with 50 μg of either Dexo(unt), Dexo(OVA), Dexo(OVA + LPS), Dexo(OVA + CpGB) or Dexo(OVA + pIC) 1 day after receiving i.v. 10^6^ CFSE-labeled naïve OT-I cells, transgenic CD8^+^ T cells specific for the OVA_257–264_ immunodominant epitope SIINFEKL. 6 days after adoptive transfer, OT-I cells were harvested from the spleens and LNs of recipient mice and analyzed by flow cytometry for proliferation and expression of markers indicative of the acquisition of T cell effector functions.

Proliferation of the OT-I cells, measured by flow cytometric analysis of CFSE dilution, was significantly increased both in the spleen and the LNs of those mice vaccinated with a Dexo(OVA + pIC) formulation (62.8% and 57.7% of proliferated OT-I cells in the spleens and 46.4% and 44.7% in the LNs of mice vaccinated i.v. or i.d., respectively) as compared to those mice vaccinated i.v. or i.d. with the other Dexo formulations (Fig. 2b). Both i.v. and i.d. vaccination with Dexo(OVA + pIC) also increased the percentage of CD62L^−^CD44^+^ effector memory OT-I cells up to 14.1% and 12.1%, respectively, in the spleens and up to 21.4% and 20.0%, respectively, in the LNs (Fig. 2c). Acquisition of effector functions by adoptively transferred OT-I cells was further confirmed by their expression of IFNγ (Fig. S1).

These results prove that in an OT-I adoptive transfer model of vaccination, Dexo(OVA + pIC), i.e. Dexo generated from BMDCs cultured in the presence of OVA and matured by the TLR-3 agonist poly(I:C), efficiently stimulate antigen-specific CD8^+^ T cells to expand and to acquire effector functions. The overall immune stimulatory effect of Dexo(OVA + pIC) on OT-I cells was significantly higher than the effect of the Dexo vaccines purified from LPS- or CpG-B-matured BMDCs, demonstrating the higher efficacy of TLR-3 stimulation for the induction of cellular immune responses by Dexo vaccines. Interestingly, comparison of the i.v. and i.d. routes of administration showed that, unlike usual vaccine formulations, Dexo vaccines are equally immunogenic when administered either i.v. or i.d.

### Increased induction of OVA-specific endogenous CD4^+^and CD8^+^T cell immune responses by Dexo(OVA+pIC) vaccination

**Figure 3:**
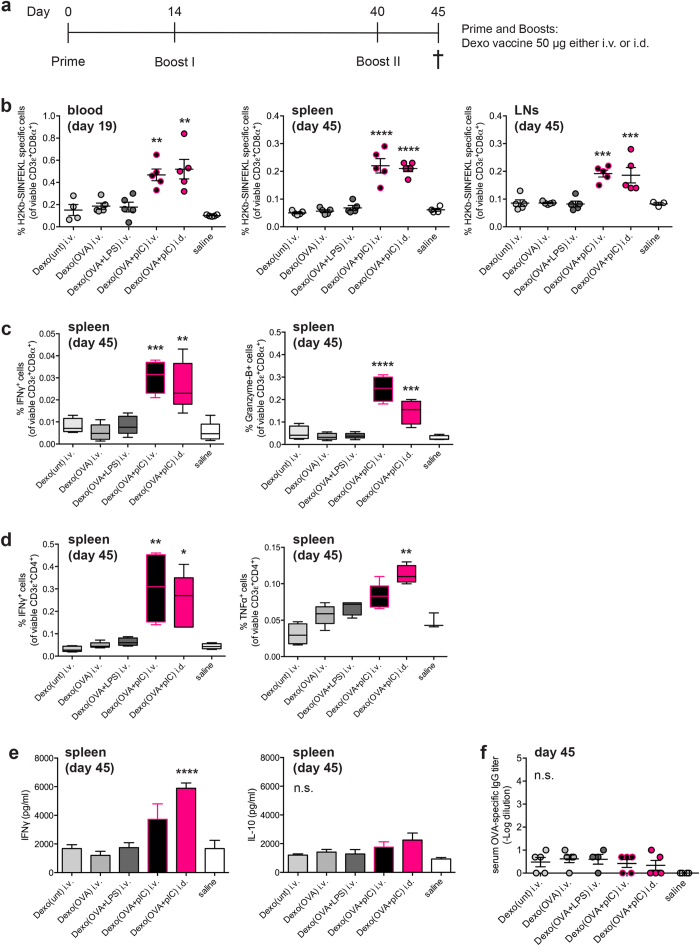
Vaccination with exosomes from OVA-loaded and poly(I:C)-activated DCs induces the expansion and acquisition of effector functions of endogenous OVA-specific CD4^+^ and CD8^+^ T cells with negligible OVA-specific antibody titers *in vivo*. (**a**) Wild-type mice were vaccinated with 50 μg of the indicated Dexo formulation or saline on day 0 (prime), 14 (boost I) and 40 (boost II). On day 45, spleens and LNs were collected to analyze OVA-specific T cell responses, and blood was sampled to measure the titer of OVA-specific IgG antibodies in the serum. (**b**) Pentamer staining and flow cytometric analysis were used to measure the frequency of SIINFEKL-specific CD8^+^ T lymphocytes in the blood of vaccinated mice on day 19 (left) or at the end of the experimental time on day 45 in the spleen (middle) and LNs (right) in the population of viable CD3ε^+^CD8α^+^ cells. (**c**) Splenocytes from vaccinated mice were collected on day 45 to measure acquisition of effector functions by SIINFEKL-specific CD8^+^ T lymphocytes as detected by intracellular staining for IFNγ (left) and Granzyme-B (right) and flow cytometric analysis. (**d**) Splenocytes from vaccinated mice were collected on day 45 to measure acquisition of effector functions by OVA-specific CD4^+^ T lymphocytes as detected by intracellular staining for IFNγ (left) and TNFα (right) and flow cytometric analysis. (**e**) Splenocytes from vaccinated mice were collected on day 45 to measure IFNγ or IL-10 secreted in the cell supernatant by ELISA upon restimulation in the presence of OVA. (**f**) Blood from vaccinated mice was collected on day 45 and the titer of OVA-specific IgG antibodies in the serum was measured by ELISA. Data represent mean ± SEM from 2 independent experiments (N = 10). Statistical analysis was performed by one-way ANOVA and Bonferroni *post*-*hoc* test correction. ^*^P < 0.05 ^**^P < 0.01, ^***^P < 0.001, ^****^P < 0.0001 and n.s. = not significant for comparisons of Dexo(OVA + pIC) administered i.v. or i.d. with Dexo(OVA + LPS).

Although the OT-I adoptive transfer model of vaccination showed a significant increase in the ability of Dexo(OVA + pIC) vaccination to activate OVA-specific CD8^+^ T cells compared to other Dexo formulations, we wanted to test if the same vaccine was also able to initiate an endogenous OVA-specific Th1 response. Since other groups have previously used i.v. administration of Dexo-based vaccines, and Dexo formulations obtained with LPS-matured DCs have previously been shown to elicit beneficial antigen-specific immune responses^16^,^21^, we sought to compare our Dexo(OVA + pIC) formulation administered either i.v. or i.d. to i.v. administration of either Dexo(unt), Dexo(OVA) or Dexo(OVA + LPS). To do so, the immune system of C57BL/6 recipient mice was first primed on day 0 and then boosted twice on day 14 and 40 with 50 μg of the indicated Dexo vaccine, as shown in Fig. 3a.

We monitored the frequency of OVA_257–264_ (SIINFEKL)-specific CD8^+^ T cells throughout the experimental period by H-2Kb/SIINFEKL pentamer staining and flow cytometric analysis of circulating lymphocytes on day 19 and of splenocytes and LN cells on day 45. As compared to vaccination with the other Dexo vaccine formulations tested, vaccination with Dexo(OVA + pIC) resulted in significantly increased frequencies of circulating SIINFEKL-specific CD8^+^ T lymphocytes already 19 days after priming, with up to 0.47% and 0.52% of pentamer positive viable CD3^+^ CD8^+^ T cells upon i.v. and i.d. vaccination, respectively (Fig. 3b, left). The difference in the frequencies of SIINFEKL-specific CD8^+^ T cells between mice administered with Dexo(OVA + pIC) and those administered with the other Dexo vaccines was even more significant at the end of the experimental timeline 45 days after priming. At this time point, in the spleens of the mice vaccinated with i.v. or i.d. Dexo(OVA + pIC) we detected 0.22% and 0.21% of pentamer positive viable CD3^+^ CD8^+^ T lymphocytes, respectively, as compared to 0.0062% detected in the spleens of mice vaccinated with i.v. Dexo(OVA + LPS) (Fig. 3b, middle). In the LNs, Dexo(OVA + pIC) vaccination induced 0.19% SIINFEKL-specific CD8^+^ T lymphocytes when performed either i.v. or i.d., as compared to only 0.082% upon i.v. administration of Dexo(OVA + LPS) (Fig. 3b, right).

The acquisition of cytotoxic effector functions by OVA-specific CD8^+^ T lymphocytes in Dexo vaccinated mice was measured after splenocytes and LN cells were restimulated for 6 hr in the presence of SIINFEKL peptide. Cytotoxic effector functions were evaluated by intracellular staining of IFNγ and Granzyme-B and flow cytometric analysis of CD3^+^ CD8^+^ lymphocytes. In mice vaccinated with i.v. or i.d. Dexo(OVA + pIC), we measured a significantly higher frequency of IFNγ^+^ or Granzyme-B^+^ CD3^+^ CD8^+^ splenocytes (Fig. 3c). 0.031% and 0.026% of IFNγ^+^ CD3^+^ CD8^+^ splenocytes were detected in mice vaccinated with Dexo(OVA + pIC) either i.v. or i.d., respectively, as compared to 0.0085% measured in mice receiving i.v. Dexo(OVA + LPS) vaccination (Fig. 3c, left). While in the spleens of mice vaccinated with i.v. Dexo(OVA + LPS) we detected 0.038% of Granzyme-B^+^ CD3^+^ CD8^+^ T cells, in mice vaccinated with i.v. or i.d. Dexo(OVA + pIC) we measured 0.25% and 0.15% of Granzyme-B^+^ CD3^+^ CD8^+^ splenocytes, respectively (Fig. 3c, right). Similar trends were also observed in the LNs of vaccinated mice, with i.v. or i.d. administration of Dexo(OVA + pIC) resulting in a significantly higher frequency of IFNγ^+^ or Granzyme-B^+^CD3^+^CD8^+^ T lymphocytes as compared to the other Dexo formulations tested (Fig. S2a).

To evaluate acquisition of the Th1 phenotype by OVA-specific CD4^+^ T lymphocytes, splenocytes and LN cells from vaccinated mice were restimulated for 6 hr in the presence of OVA, stained for IFNγ and TNFα and analyzed by flow cytometry. In mice vaccinated with i.v. or i.d. Dexo(OVA + pIC), we measured a significantly higher frequency of IFNγ^+^ or TNFα^+^CD3^+^CD4^+^ lymphocytes as compared to mice vaccinated with the other Dexo formulations (Fig. 3d). 0.31% and 0.25% of IFNγ^+^CD3^+^CD4^+^ splenocytes were measured in mice vaccinated with Dexo(OVA + pIC) either i.v. or i.d., respectively, as compared to 0.064% detected in mice receiving i.v. Dexo(OVA + LPS) vaccination (Fig. 3d, left). Vaccination with Dexo(OVA + pIC) also significantly raised TNFα^+^CD3^+^CD4^+^ splenocytes up to 0.083% and 0.11% upon i.v. or i.d. administration, respectively (Fig. 3d, right). Similarly to the spleen, the frequencies of IFNγ^+^ and TNFα^+^CD3^+^CD4^+^ lymphocytes were also significantly higher in the LNs of mice vaccinated with Dexo(OVA + pIC) (Fig. S2b). Significantly increased expression and release of the Th1 cytokines IFNγ and TNFα by the splenocytes of mice vaccinated with i.v. or i.d. administration of Dexo(OVA + pIC) was further confirmed by ELISA (Fig. 3e, left, and Fig. S2c). Secretion of IL-10, instead, was limited, and differences were not significant among the different vaccine treatments tested, thus confirming skewing towards a Th1 immune response rather than Th2 by vaccination with Dexo(OVA + pIC) (Fig. 3e, right).

Even though vaccination with Dexo(OVA + pIC) stimulated a greater OVA-specific Th1 immune response as compared to Dexo(unt), Dexo(OVA) and Dexo(OVA + LPS), none of the Dexo formulations tested was able to induce the production of OVA-specific antibodies (Fig. 3f), thus confirming the absence of free intact OVA contaminating the Dexo formulations, rather suggesting the presence of OVA or OVA-derived peptides encapsulated inside exosomes or bound to MHC class I and class II molecules on the surface of exosomes, as previously shown by other groups^16^,^30^,^31^.

These data indicate that poly(I:C)-induced maturation of DCs cultured in the presence of an antigen improves the ability of DC-released Dexo to stimulate antigen-specific cellular rather than humoral immune responses. In particular, vaccination with Dexo(OVA + pIC) stimulates the expansion and activation of endogenous OVA-specific CD4^+^ and CD8^+^ T cells, skewing the former towards the pro-inflammatory Th1 phenotype and stimulating the latter to acquire cytotoxic functions more potently than Dexo derived by DC maturation induced by TLR ligands previously used by other groups, most notably LPS^16^.

### Isolation of DC-derived exosomes containing B16F10 antigens and their use as a therapeutic vaccine in the B16F10 melanoma model

**Figure 4:**
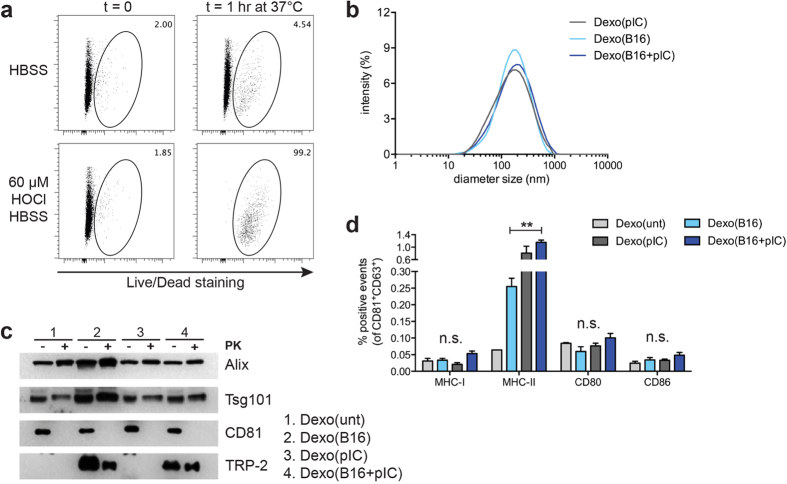
HOCl-oxidized B16-F10 melanoma cells can be used as a source of tumor antigens for the production of DC exosomes containing melanoma-derived epitopes. (**a**) B16-F10 melanoma cells were resuspended in 60 μM HOCl HBSS buffer and incubated at 37 °C for 1 hr to induce oxidation of the tumor cells. After incubation, cells were stained with a viability dye and analyzed by flow cytometry. As controls, viability of B16-F10 cells before incubation (t = 0) or B16-F10 cells resuspended in HBSS buffer was also analyzed. Numbers indicate the frequency of dead cells gated in the total population of B16-F10 cells. (**b**) Dexo were purified from the supernatant of DCs preincubated with oxidized B16-F10 obtained as in (**a**) with or without poly(I:C) (Dexo(B16 + pIC) and Dexo(B16), respectively) or with poly(I:C) only as a control (Dexo(pIC)) following the described protocol for exosomes isolation. After purification, the size of Dexo was measured by DLS analysis. (**c**) Presence of the exosome-specific markers Alix (100 kDa) and Tsg101 (46 kDa) (intravesicular) and CD81 (26 KDa) (vesicle membrane) and of the full-length melanoma-derived protein TRP-2 (59 kDa) was detected by western blot analysis of Dexo(unt), Dexo(B16), Dexo(pIC) and Dexo(B16 + pIC) digested or not with proteinase K to confirm purification of *bona-fide* exosomes and exosomal localization of the antigens. (**d**) Surface staining for MHC-I, MHC-II, CD80 and CD86 and flow cytometric analysis of Dexo(unt), Dexo(B16), Dexo(pIC) and Dexo(B16 + pIC). Percentages represent the frequency of positive events among CD81^+^ CD63^+^ particles. Data in (**d**) represent mean ± SEM from 2 independent experiments (N = 6). Statistical analysis was performed by one-way ANOVA and Bonferroni *post*-*hoc* test correction to compare Dexo(B16), Dexo(pIC) and Dexo(B16 + pIC). ^**^P < 0.01 and n.s. = not significant.

Dexo vaccination had previously entered clinical trials for the treatment of melanoma patients, resulting in poor therapeutic benefit^18^. We therefore sought to test whether our poly(I:C)-based Dexo vaccine could improve the efficacy of Dexo vaccination in a clinically relevant setting of therapeutic vaccination in melanoma-bearing mice, taking advantage of the B16F10 model. Based on the higher immunogenicity of Dexo produced with poly(I:C) as compared to Dexo produced with either LPS or CpG-B as shown in Figs 2 and 3, we chose to only utilize poly(I:C) for the production of a Dexo vaccine that could be compared to a Dexo formulation resembling those tested in the clinical trials produced from DCs cultured in the presence of tumor antigens but without adjuvants. To produce DC-derived exosomes carrying native B16F10 antigens instead of the model antigen OVA, we adopted the protocol developed by Chiang *et al.* to pulse patient-derived DCs with autologous tumor antigens^32^,^33^. To do so, B16F10 cells were incubated with 60 μM HOCl buffer to induce oxidation-dependent necrosis of the tumor cells, thus allowing the release of melanoma antigens in an immunogenic fashion (Fig. 4a). Following the same procedure utilized for the production of our OVA-containing Dexo vaccine formulations, HOCl-oxidized B16F10 cells were cultured together with BMDCs in the presence or not of poly(I:C) to harvest Dexo(B16 + pIC) or Dexo(B16), respectively. As an experimental control, we also purified exosomes from DCs matured with poly(I:C) in the absence of oxidized B16F10 cells (Dexo(pIC)).

DLS and western blot analysis of Dexo(B16), Dexo(pIC) and Dexo(B16 + pIC) were used to confirm the presence of vesicles with size (30–150 nm in diameter) and markers (Alix, Tsg101 and CD81) indicative of *bona-fide* exosomes (Fig. 4b,c). To prove exosomal packaging of melanoma antigens in Dexo(B16) and Dexo(B16 + pIC), we probed Dexo(B16), Dexo(pIC) and Dexo(B16 + pIC) samples for the B16F10-derived tyrosinase-related protein 2 (TRP-2). To provide information about the localization of the melanoma-derived TRP-2, Dexo samples were digested with proteinase K (PK) or left untreated before western blot analysis. While detection of the intra-exosomal markers Alix and Tsg101 was not affected by digestion with PK, the exosomal membrane-spanning antigen CD81 could not be revealed after treatment of the exosomes with PK, indicating its export to the extra-vesicular space. Similarly to Alix and Tsg101, TRP-2 was also protected from the enzymatic activity of PK, indicating its intra-exosomal localization, in this case at its expected full-length molecular weight, as contrasted to that observed in Fig. 1c with OVA (Fig. 4c).

Differences among Dexo(B16), Dexo(pIC) and Dexo(B16 + pIC) could be detected by flow cytometry in their surface expression of the antigen-presenting complex MHC-II, which was significantly enriched especially on Dexo(B16 + pIC), as a consequence of strong DC maturation upon incubation with oxidized tumor cells in the presence or not of poly(I:C) (Fig. 4d).

**Figure 5:**
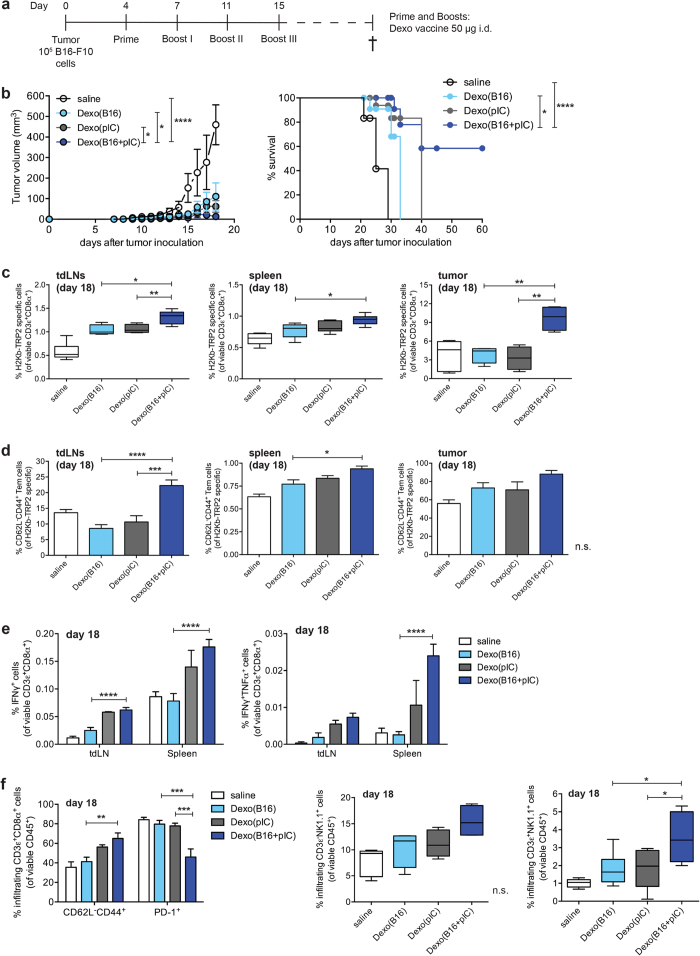
Therapeutic vaccination with tumor cell antigen-loaded exosomes from poly(I:C)-activated DCs significantly reduces the growth of B16-F10 tumors and improves the survival of tumor-bearing mice by activating melanoma-specific CD8^+^ T cells and promoting tumor infiltration of cytotoxic cells. (**a**) B16-F10 melanoma cells were inoculated subcutaneously between the scapulae of C57BL/6 mice on day 0. To target the tdLNs, recipient mice were vaccinated i.d. in the frontal footpads with 50 μg of the indicated Dexo formulation or saline on day 4 (prime), 7 (boost I), 11 (boost II) and 15 (boost III). (**b**) Tumor volumes (left) and survival rate (right) were measured from day 0 to day 18 and from day 0 to day 60 after tumor inoculation, respectively. (**c**) Frequencies of TRP-2_180–188_ (SVYDFFVWL)-specific CD8^+^ T lymphocytes in the population of viable CD3ε^+^CD8α^+^ cells harvested on day 18 from the tdLNs (left), spleen (middle) and tumor mass (right). (**d**) Frequencies of CD62L^-^CD44^+^ effector memory TRP2-specific CD8^+^ T lymphocytes harvested on day 18 from the tdLNs (left), spleen (middle) and tumor mass (right). (**e**) Acquisition of effector functions by B16-F10-specific CD8^+^ T lymphocytes as indicated by intracellular staining of IFNγ and TNFα and flow cytometric analysis. (**f**) Frequencies of tumor-infiltrating CD62L^-^CD44^+^ effector memory and exhausted PD-1^+^ CD8^+^T lymphocytes in the population of total viable CD45^+^CD3ε^+^CD8α^+^ cells (left). Frequencies of tumor-infiltrating NK cells (middle) and NK-T cells (right) among viable CD45^+^ CD3ε^-^NK1.1^+^ or CD45^+^ CD3ε^+^ NK1.1^+^ cells, respectively. Data represent mean ± SEM from 2 independent experiments (N = 15). Statistical analysis of tumor volumes in (**b**) was performed by unpaired t test comparing Dexo(B16 + pIC) group with either saline, Dexo(B16) or Dexo(pIC) group. ^*^P < 0.1; ****P < 0.0001. Statistical analysis of survival rates in (**b**) was performed by Log-rank Mantel-Cox test. ^*^P < 0.05; ^****^P < 0.0001. Statistical analysis of data in (**d**–**f**) was performed by one-way ANOVA and Bonferroni *post-hoc* test correction. ^*^P < 0.05, ^**^P < 0.01, ^***^P < 0.001, ^****^P < 0.0001 and n.s. = not significant for comparisons of Dexo(B16 + pIC) with Dexo(B16) and Dexo(pIC).

We proceeded to test whether therapeutic i.d. vaccination with Dexo(B16) or Dexo(B16 + pIC) could result in any beneficial outcome in B16F10 tumor-bearing mice. Vaccination with Dexo(pIC) was chosen as a control to test the melanoma specificity of the immune responses raised by Dexo(B16) and Dexo(B16 + pIC) vaccination. For this purpose, 10^5^ B16F10 cells were inoculated subcutaneously (s.c.) between the scapulae in C57BL/6 mice on day 0 and left to engraft and grow for 4 days. On day 4, 7, 11 and 15, mice were either administered with saline or vaccinated with 50 μg of Dexo(B16), Dexo(pIC) or Dexo(B16 + pIC) i.d. in the frontal footpads to improve vaccine efficacy by targeting the immune cells of the tdLNs^34^. Tumor growth was measured until 18 days after inoculation of the B16F10 cells, when the anti-melanoma immune response in the tumor mass, spleen and tdLNs was also analyzed. To determine the survival rate, vaccinated tumor-bearing mice were also kept until death occurred or euthanasia was necessary due to ethical reasons. In the case of mice surviving with no sign of debilitating sickness, experiments were ended 60 days after implantation of the tumor cells (Fig. 5a).

Interestingly, all of the Dexo formulations tested were able to significantly reduce the growth of B16F10 tumors as compared to vehicle administration. Most importantly, vaccination with Dexo(B16 + pIC) proved the combination of loading with melanoma antigens and stimulation with poly(I:C) for DC-derived exosome production beneficial, resulting in significant reduction of tumor growth not only as compared to saline treatment but also as compared to vaccination with either Dexo(B16) or Dexo(pIC) (Fig. 5b, left). The Dexo vaccines also improved the survival rate of B16F10 tumor-bearing mice and, even though therapeutic vaccination with Dexo(B16 + pIC) could not prevent B16F10 tumors from growing, it did slow tumor progression, improving long-term survival of vaccinated mice, with 58% of Dexo(B16 + pIC)-vaccinated mice showing no sickness symptoms at the end of the experimental time of 60 days (Fig. 5b, right).

To dissect the immunological effects behind the benefits provided by Dexo vaccination, we chose the melanoma-derived protein TRP-2 as a representative antigen to analyze the immune response elicited against B16F10 tumor cells upon Dexo vaccination. The frequency of TRP-2_180–188_ (SVYDFFVWL)-specific CD3^+^CD8^+^ T lymphocytes was measured in the cells from the tdLNs, spleens and tumor masses of vaccinated mice 18 days after tumor cells were inoculated by pentamer staining and flow cytometry. All of the Dexo vaccine formulations tested were able to increase the frequency of TRP2-specific CD8^+^ T cells as compared to saline treatment, but only vaccination with Dexo(B16 + pIC) resulted in significantly increased TRP2-specific CD8^+^ T lymphocytes as compared to saline and to the other Dexo formulations, not only in the tdLNs (1.32%) and spleens (0.94%) but also in the tumor masses of treated mice (9.7%) (Fig. 5c). Of note, the TRP2-specific CD8^+^ T cells induced by vaccination with Dexo(B16 + pIC) also showed increased expression of the effector memory phenotype markers CD62L^-^CD44^+^, with 22.28%, 0.94% and 88.1% of CD62L^-^CD44^+^TRP2-specific CD8^+^ T lymphocytes detected in the tdLNs, spleens and tumor masses, respectively (Fig. 5d). The acquisition of effector functions by B16F10-specific CD8^+^ T cells was further confirmed by restimulating *ex vivo* for 6 hr tdLN cells and splenocytes from vaccinated mice with oxidized B16F10 cells (obtained as in Fig. 4a) and staining for intracellular IFNγ and TNFα for flow cytometric analysis. Cells from mice receiving Dexo(B16 + pIC) vaccination responded more strongly to B16F10 antigen-specific restimulation, resulting in 0.062% and 0.176% of IFNγ^+^CD3^+^CD8^+^ T lymphocytes and 0.0073% and 0.024% of IFNγ^+^TNFα^+^CD3^+^CD8^+^ T cells in the tdLNs and spleens, respectively (Fig. 5e).

Interestingly, tdLN-targeted administration of our Dexo vaccines resulted in favorable recruitment and stimulation of cytotoxic CD45^+^ cells infiltrating the tumors, especially when Dexo(B16 + pIC) vaccine was administered. In the population of tumor-infiltrating CD8^+^ T lymphocytes, effector memory CD62L^-^CD44^+^ cells were significantly increased (65.1%), while exhausted PD-1^+^ cells were significantly reduced (45.95%), in mice vaccinated with Dexo(B16 + pIC) (Fig. 5f, left). Even though the difference was not statistically significant, also NK cells showed a trend toward increased frequency in the population of hematopoietic cells infiltrating the tumors of Dexo(B16 + pIC)-treated mice (15.5%) as compared to mice administered with saline or the other Dexo formulations (Fig. 5f, middle). NK-T cells, instead, were significantly more represented among CD45^+^ tumor-infiltrating cells (3.57%) upon Dexo(B16 + pIC) vaccination (Fig. 5f, right).

**Figure 6:**
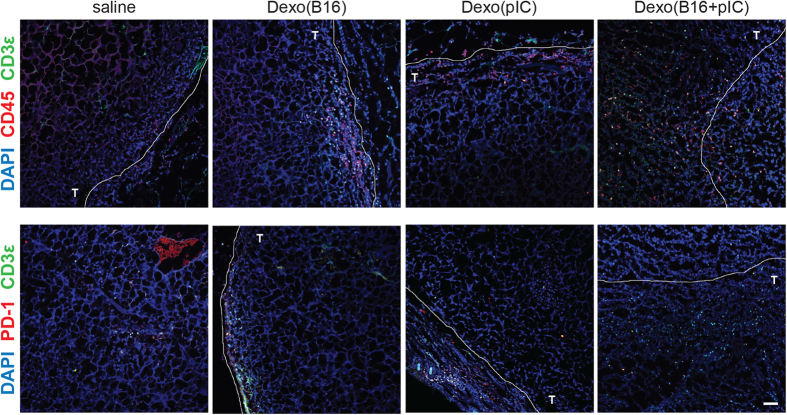
Vaccination with tumor cell antigen-loaded exosomes from poly(I:C)-activated DCs induces massive recruitment and infiltration of the tumor mass by PD-1^–^ lymphocytes. Tumor sections from C57BL/6 mice vaccinated as indicated in Fig. 5a with the indicated Dexo formulation or saline and euthanized on day 18 were stained for either CD45 and CD3ε (top) or CD45 and PD-1 (bottom) and imaged by confocal microscopy. T = intratumoral area. Representative images from 2 independent experiments are shown (N = 15). Scale bar = 50 μm.

Confocal microscopy of tumor sections revealed that even though all the Dexo vaccines tested were capable of recruiting CD45^+^ hematopoietic cells and, especially, CD3^+^ cells at the tumor site as compared to saline treatment, Dexo(B16 + pIC) vaccination induced greater hematopoietic recruitment and, most importantly, infiltration of the tumor mass by lymphocytes (Fig. 6, top). Confirming the flow cytometry data described in Fig. 5f, the majority of tumor-infiltrating CD3^+^ cells in the tumors harvested from Dexo(B16 + pIC)-vaccinated mice was also PD-1^−^ (Fig. 6, bottom).

These data indicate that the purification of exosomes containing antigens from a specific tumor cell type, in our case the B16F10 melanoma cells, is feasible. Moreover, tdLN-targeted therapeutic vaccination with exosomes derived from DCs loaded with B16F10 antigens and matured with poly(I:C) shows a beneficial capacity of stimulating B16F10-specific effector CD8^+^ T cells and recruiting effector T lymphocytes, NK and NK-T cells to the tumor site resulting in smaller tumors and prolonged survival of tumor-bearing mice.

## Discussion

DC-derived exosomes represent an attractive alternative to DC-based vaccines for immunotherapy in cancer patients^5^,^7^,^20^. Dexo display immunologically active components derived from DCs but, as vesicles, they constitute a non-living cell-free vaccine tool. Importantly, pre-clinical use of Dexo vaccines in murine tumor models, especially melanoma, has shown the ability of Dexo to stimulate strong antitumoral adaptive immunity, resulting in the suppression of tumor growth^11^,^12^,^13^,^14^,^15^,^16^,^17^. On the basis of these promising pre-clinical results, clinical trials were conducted to test therapeutic vaccination with Dexo in patients with late-stage melanoma and non-small cell lung carcinoma. For vaccination in the human subjects, Dexo were produced from autologous DCs and were loaded either directly or indirectly with tumor peptides in the absence of any adjuvants. Even though such clinical trials proved the feasibility of large-scale production of antigen-loaded autologous DC-derived exosomes for use in humans, they failed to prove therapeutic benefits of the vaccination protocol since tumor-specific immunity was elicited only in a limited number of the treated patients^18^,^19^,^20^.

Other groups have tried to improve the immunogenicity of Dexo vaccines by developing a formulation that could more effectively induce anti-tumor cytotoxic responses. Use of Dexo rather than tumor cell-derived exosomes and their loading with intact antigens rather than with peptides was shown to induce more potent antitumor immunity related to the activation of a broader repertoire of antigen-specific immune cell clones *in vivo*. Moreover, incubation with TLR ligands, either during exosome production (LPS) or after exosome purification (Pam_3_ or poly(I:C)), resulted in the induction of beneficial pro-inflammatory Th1 responses both *in vitro* and *in vivo* in tumor-bearing animals^16^,^17^,^21^,^25^.

The goal of this study was to exploit the intrinsic properties of DCs to take up and process antigens and sense danger signals to mount strong antigen- and adjuvant-tailored immune responses. To do so, we produced exosomes from antigen-loaded DCs that were matured with different TLR ligands, to explore the effects of DC stimulation with different danger signals on the immunogenicity of the exosomes that they secrete. As TLR ligands for DC maturation, we chose TLR agonists that could result in efficient stimulation of Th1 responses both in mice and in humans and that could be easily transferrable to a clinical setting, such as the TLR-3 agonist poly(I:C) and the TLR-9 agonist CpG-B^25^,^27^,^28^,^29^. Future studies will also explore alternative and possibly more potent TLR agonists for the production of novel Dexo-based vaccine formulations, either as single adjuvant or in combination. Among other candidates, the TLR-4 ligand monophosphoryl lipid A (MPLA) component of LPS is of particular interest as it has shown a promising profile in terms of both safety and immunogenicity in several clinical trials for cancer immunotherapy^35^.

Our results show that exosomes purified from the supernatant of DCs loaded with the model antigen OVA and matured with poly(I:C) (Dexo(OVA + pIC)) are significantly more potent activators of antigen-specific Th1 responses than exosomes purified from CpG-B- or LPS-matured DCs, both in an OT-I transfer model of vaccination and in an endogenous model of vaccination. While adoptively transferred OT-I cells responded to Dexo(OVA + pIC) vaccination with vigorous proliferation but only modest acquisition of effector phenotype (CD62L^−^CD44^+^) and functions (IFNγ expression) (Fig. 2), endogenous OVA-specific CD4^+^ and CD8^+^ T cells strongly expanded and synthesized Th1 cytokines upon Dexo(OVA + pIC) administration (Fig. 3). Interestingly, vaccination with Dexo(OVA + pIC) elicited an anti-OVA cellular response in the absence of detectable OVA-specific antibodies (Fig. 3f). The lack of humoral response is consistent with the measurement of no intact OVA antigen being present in the Dexo vaccines we produced, and rather indicates the presence of OVA or OVA-derived peptides encapsulated inside exosomes or bound to MHC class I and class II molecules on the surface of exosomes that could be transferred or presented to APCs and T cells initiating the antigen-specific immune response *in vivo*, as previously published^10^,^16^,^30^,^31^.

The efficient stimulation of antigen-specific cellular responses by a poly(I:C)-based Dexo vaccine is highly attractive in the context of therapeutic vaccination for cancer immunotherapy. We thus compared the outcome of a therapeutic vaccination protocol with Dexo produced from B16F10 antigen-loaded DCs with or without poly(I:C)-driven maturation. The B16F10-Dexo vaccines were produced by loading the DCs with a B16F10 cell lysate obtained by oxidation-induced necrosis, rather than with a single melanoma antigen, to stimulate a polyclonal immune response against the tumor cells and provide additional cell-derived danger signals to the DCs other than poly(I:C). In fact, DC pulsing with oxidized tumor cells has been previously shown to enhance the immunogenicity of tumor lysates, resulting in more potent T cell responses in murine models of ovarian cancer and in effective broad anti-tumor immunity and therapeutic beneficial effects in ovarian cancer patients^32^,^33^. Even though we cannot exclude that a part of the exosomes we purify after co-culturing the DCs with oxidized B16F10 cells is derived from the tumor cells rather than from the DCs, our data show that we are able to purify exosomal vesicles containing antigens from the melanoma cells (like TRP-2), so that they can act as antigen carriers upon vaccination (Fig. 4). In contrast to the results with Dexo from OVA-pulsed DCs, we did find full-length TRP-2 in the Dexo from DCs pulsed with oxidized B16F10 cells (Figs 1c and 4c). This may be due to differential efficiency of the antigen processing mechanisms within the DCs between two protein antigens, to differences in the processing mechanisms of free versus cellular antigens, or to presence of exosomes derived from the tumor cells themselves. The intact TRP-2 antigen was not carried over as soluble contaminant, in that it was protected from degradation by extravesicular proteinase K, thus demonstrating its presence within the Dexo particles (Fig. 4c). More importantly, significant enrichment of MHC-II molecules could be detected on the surface of Dexo(B16 + pIC) particles, indicating not only that treatment of exosome-secreting DCs with oxidized tumor cells and poly(I:C) provides strong maturation signals but also that the secreted Dexo inherit improved antigen-presenting and immune stimulatory capabilities from their cells of origin (Fig. 4d).

To maximize the therapeutic effect of Dexo vaccines, we chose to administer Dexo intradermally only at those anatomic sites suitable to target the vaccine to the immune cells of the tdLNs, as it was previously published that targeting the tdLNs provides a more beneficial outcome than targeting the non-tdLNs^34^,^36^,^37^.

Indeed, tdLN-targeted vaccination with Dexo(B16 + pIC) resulted in reduced tumor growth and prolonged survival of tumor-bearing mice, associated with significantly higher frequencies of melanoma-specific cytotoxic CD8^+^ T cells and of CD62L^-^CD44^+^ effector memory CD8^+^ T, NK and NK-T cells and lower frequencies of PD-1^+^ exhausted CD8^+^ T cells infiltrating the tumors as compared to all the other Dexo vaccine formulations we tested (Figs 5 and 6). Of note, only vaccination with Dexo(B16 + pIC) stimulated massive infiltration of hematopoietic cells, and in particular of PD-1^-^ lymphocytes, into the tumor mass of treated mice (Fig. 6).

Vaccination with Dexo(pIC), i.e. exosomes produced from poly(I:C)-matured DCs in the absence of tumor antigens, was also capable of stimulating a melanoma-specific immune response resulting in reduced tumor growth and prolonged survival as compared to vaccination with Dexo(B16). This finding is not completely unexpected, as locally administered immune adjuvants in cancer immunotherapy are known to contribute to tumor-associated inflammation leading to the enhancement of protective immunity^38^,^39^,^40^,^41^. In our experimental setting of tdLN-targeted vaccination, the simultaneous presence in the tdLNs of Dexo(pIC)- or Dexo(B16 + pIC)-delivered danger signals and of tumor-derived antigens (both draining from the tumor site and carried by Dexo) resulted in beneficial anti-cancer immunity, more effectively stimulated by vaccination with Dexo(B16 + pIC).

Previous groups showed that the adjuvants used to induce DC maturation during exosome production can bind to the exosome membrane and can thus be co-injected with the Dexo vaccine, directly activating APCs *in vivo* as a by-stander effect^21^. We provide further evidence that small amounts of poly(I:C) are in fact consistently found in poly(I:C)-Dexo vaccines after purification (Fig. S3a). Upon Dexo vaccination, poly(I:C) molecules could participate in the induction of immune cell activation and tumor growth arrest by direct engagement of TLR-3 molecules expressed by myeloid DCs and T cells and by tumor cells, respectively, even though exposure to nucleases may lead to their rapid degradation *in vivo*^42^,^43^. Nevertheless, exosomes isolated from antigen-loaded DCs matured with poly(I:C) have more potent stimulatory effects on antigen-specific CD8^+^ T lymphocytes than equivalent doses of free poly(I:C) and such effects cannot be abrogated by specifically blocking the TLR-3, thus excluding that the immunogenic effect of poly(I:C)-based Dexo depends exclusively on the adjuvant molecules contaminating each Dexo formulation (Fig. S3b). We instead speculate that the DC-derived molecular components of Dexo, such as MHC complexes, co-stimulatory molecules, adhesion molecules and pro-inflammatory signals, together with antigenic epitopes as well as adjuvant molecule carry-over all concomitantly participate in positively affecting the immunogenic properties of a Dexo vaccine.

In summary, we provide evidence that exosomes produced from antigen-loaded DCs matured with the TLR-3 agonist poly(I:C) are more potent immunogens than exosomes produced with LPS for TLR-4 or CpG-B for TLR-9. We show that the purification of DC-derived exosomes carrying epitopes from melanoma cells is feasible and is able to produce potent antitumoral immunity, leading to tumor growth control in 58% of the animals treated. This approach is potentially transferrable to any type of clinically relevant tumor cell for the development of potent tumor-tailored Dexo vaccines.

## Methods

### Reagents

Chemicals and reagents were purchased from Sigma-Aldrich (Buchs, Switzerland), unless specifically indicated. RPMI 1640 GlutaMAX, IMDM GlutaMAX and DMEM GlutaMAX media, FBS, penicillin/streptomycin, 2-Mercaptoethanol and HBSS buffer were purchased from Life Technologies (Carlsbad, CA). Chemically defined *X-VIVO* 15 medium was from Lonza (Basel, Switzerland). Abs for western blot were purchased from Sigma-Aldrich or Bio-Rad (Hercules, CA). Live/dead fixable cell viability reagents for flow cytometry were from Life Technologies. PE-labeled H-2Kb/SIINFEKL and H-2Kb/TRP-2_180–188_ (SVYDFFVWL) pentamers were purchased from Proimmune (Oxford, UK). Abs used in flow cytometry were from eBioscience (Vienna, Austria) or BioLegend (Lucerne, Switzerland).

### Production of OVA-loaded DC-derived exosomes (Dexo)

Bone-marrow derived DCs (BMDCs) were generated by culturing total bone marrow cells from 8-10 week old female C57BL/6 mice (Harlan Laboratories, Gannat, France) in RPMI 1640 GlutaMAX medium supplemented with 10% FBS, 100 IU/mL penicillin-streptomycin, 50 μM 2-Mercaptoethanol and 20 ng/mL recombinant murine GM-CSF (PeproTech, Rocky Hill, NJ) at 37 °C 5% CO_2_. On day 3, a volume of complete medium equal to the original culture volume was added to the cells. On day 6, BMDCs were harvested, washed in PBS and cultured for OVA antigen loading. To produce Dexo(unt), BMDCs were cultured in complete medium at 37 °C 5% CO_2_ for 12 hr. To produce OVA-loaded Dexo, BMDCs were cultured in complete medium supplemented with 300 μg/mL endo-grade chicken OVA (Hyglos, Bernried am Starnberger See, Germany) at 37 °C 5% CO_2_ for 12 hr. After 12 hr, BMDCs were harvested, washed extensively in PBS to eliminate serum and free OVA and cultured in *X-VIVO* 15 medium for the production of Dexo(unt) and Dexo(OVA) or *X-VIVO* 15 medium supplemented with either 50 ng/mL LPS, 8 μg/mL CpG-B 1826 oligonucleotide (5′-TCCATGACGTTCCTGACGTT-3′ from Microsynth, Balgach, Switzerland), or 50 μg/mL poly(I:C) (*Invivo*Gen, San Diego, CA) for the production of Dexo(OVA + LPS), Dexo(OVA + CpGB) or Dexo(OVA + pIC), respectively. On day 8, exosomes were isolated by serial (ultra)centrifugation.

### Isolation of Dexo

The supernatant of BMDCs was collected and centrifuged first at 300 × *g* for 5 min at 4 °C and then at 2000 × *g* for 10 min at 4 °C, passed through 0.22 μm filters (Millipore, Billerica, MA) and subsequently spun at 110,000 × *g* for 90 min at 4 °C using a Beckman Coulter (Brea, CA) ultracentrifuge. Exosome pellets were resuspended in PBS and frozen at −80 °C prior to use.

### Mice

C57BL/6 female mice and CD45.2^+^ OT-I transgenic female mice (8 to 12 weeks of age) were obtained from Harlan Laboratories, CD45.1^+^ C57BL/6-Ly5.1 female mice (8 to 12 weeks of age) were purchased from Charles River (Saint-Germain-Nuelles, France). Animals were housed in pathogen-free conditions at the animal facility of the Ecole Polytechnique Fédérale de Lausanne and all experiments were performed in accordance with Swiss law and with approval from the Cantonal Veterinary Office of Canton de Vaud, Switzerland (Authorization number 2935).

### Production of B16F10 antigen-loaded Dexo

BMDCs were differentiated as described above. On day 6, cells were cultured in complete medium together with HOCl-oxidized B16F10 cells (1:1) at 37 °C 5% CO_2_ for 12 hr. After 12 hr, BMDCs were harvested, washed extensively in PBS and cultured in *X-VIVO* 15 medium for the production of Dexo(B16) or *X-VIVO* 15 medium supplemented with 50 μg/mL poly(I:C) for the production of Dexo(B16 + pIC). For the production of Dexo(pIC), BMDCs were only cultured with *X-VIVO* 15 medium supplemented with 50 μg/mL poly(I:C). On day 8, we proceeded to isolate Dexo by serial (ultra)centrifugation as indicated above.

### B16F10 tumor vaccination studies

On day 0, C57BL/6 mice were anesthetized with isoflurane and their back was shaved. 10^5^ B16F10 cells resuspended in 100 μL of PBS were inoculated subcutaneously between the scapulae of each mouse. On day 4, 7, 11 and 15 mice were anesthetized with isoflurane and vaccinated intradermally into the 2 frontal footpads with 50 μg of the indicated Dexo formulation. Tumors were measured with a digital caliper until day 18 after tumor inoculation, and volumes (*V*) were calculated as ellipsoids (*V* = π/6 × length × width × height). For survival rate assessment, mice were kept until death occurred or sacrifice was necessary due to sickness or tumor volumes > 1 cm^3^, as required by Swiss law. In the case of mice surviving with no sign of debilitating sickness, experiments were ended 60 days after implantation of the tumor cells.

For immunological readouts and imaging by confocal microscopy, vaccinated tumor-bearing mice were euthanized on day 18 after tumor inoculation to collect cells from the spleen, tdLNs and tumor mass. Splenocytes and tdLN cells were obtained as described above. Tumors were processed as previously published^34^.

### Statistics

Statistically significant differences between experimental groups were determined by one-way ANOVA followed by Bonferroni *post-hoc* test correction. ^*^P < 0.05, ^**^P < 0.01, ^***^P < 0.001, ^****^P < 0.0001 and n.s. = not significant.

For tumor volumes, differences between groups were determined by unpaired t test. ^*^P < 0.1; ****P < 0.0001.

For survival rate, differences between groups were determined by Log-rank Mantel-Cox test. ^*^P < 0.05; ^****^P < 0.0001.

Statistical analyses were performed using Prism software (v6.0f, GraphPad Software, San Diego, CA).

Detailed information on exosome quantification, transmission electron microscopy, western blot, mass spectrometry, vaccination studies, antigen-specific restimulation^44^, flow cytometry, B16F10 cell culture and oxidation, tumor vaccination studies, confocal microscopy imaging, TLR-3 reporter assay and^45^ data analysis can be found in the Supplementary Methods.

## Additional Information

**How to cite this article**: Damo, M. *et al.* TLR-3 stimulation improves anti-tumor immunity elicited by dendritic cell exosome-based vaccines in a murine model of melanoma. *Sci. Rep.* **5**, 17622; doi: 10.1038/srep17622 (2015).

## Supplementary Material

Supplementary Information
