# Supplementary material for: TLR-3 stimulation improves anti-tumor immunity elicited by dendritic cell exosome-based vaccines in a murine model of melanoma
Source: Sci Rep. 2015 Dec 3;5:17622. doi: 10.1038/srep17622 (PMC4668567; doi:10.1038/srep17622)
Supplement: Supplementary Information [file srep17622-s1.pdf]

TLR-3 stimulation improves anti-tumor immunity elicited by dendritic cell exosome-based vaccines in a murine model of melanoma

Martina Damo[a], David S. Wilson[a], Eleonora Simeoni[a,b], Jeffrey A. Hubbell[a,c,d,*]

[a]Institute of Bioengineering, School of Life Sciences and School of Engineering, Ecole Polytechnique Fédérale Lausanne, CH-1015 Lausanne, Switzerland;

[b]Center of PhenoGenomics, School of Life Sciences, Ecole Polytechnique Fédérale Lausanne, CH-1015 Lausanne, Switzerland;

[c]Institute for Chemical Science and Engineering, School of Basic Sciences, Ecole Polytechnique Fédérale Lausanne, CH-1015 Lausanne, Switzerland;

[d]Institute for Molecular Engineering, University of Chicago, IL 60637, USA;

[*]To whom correspondence should be addressed: jeffrey.hubbell@epfl.ch or jhubbell@uchicago.edu.

Supplementary figure-1

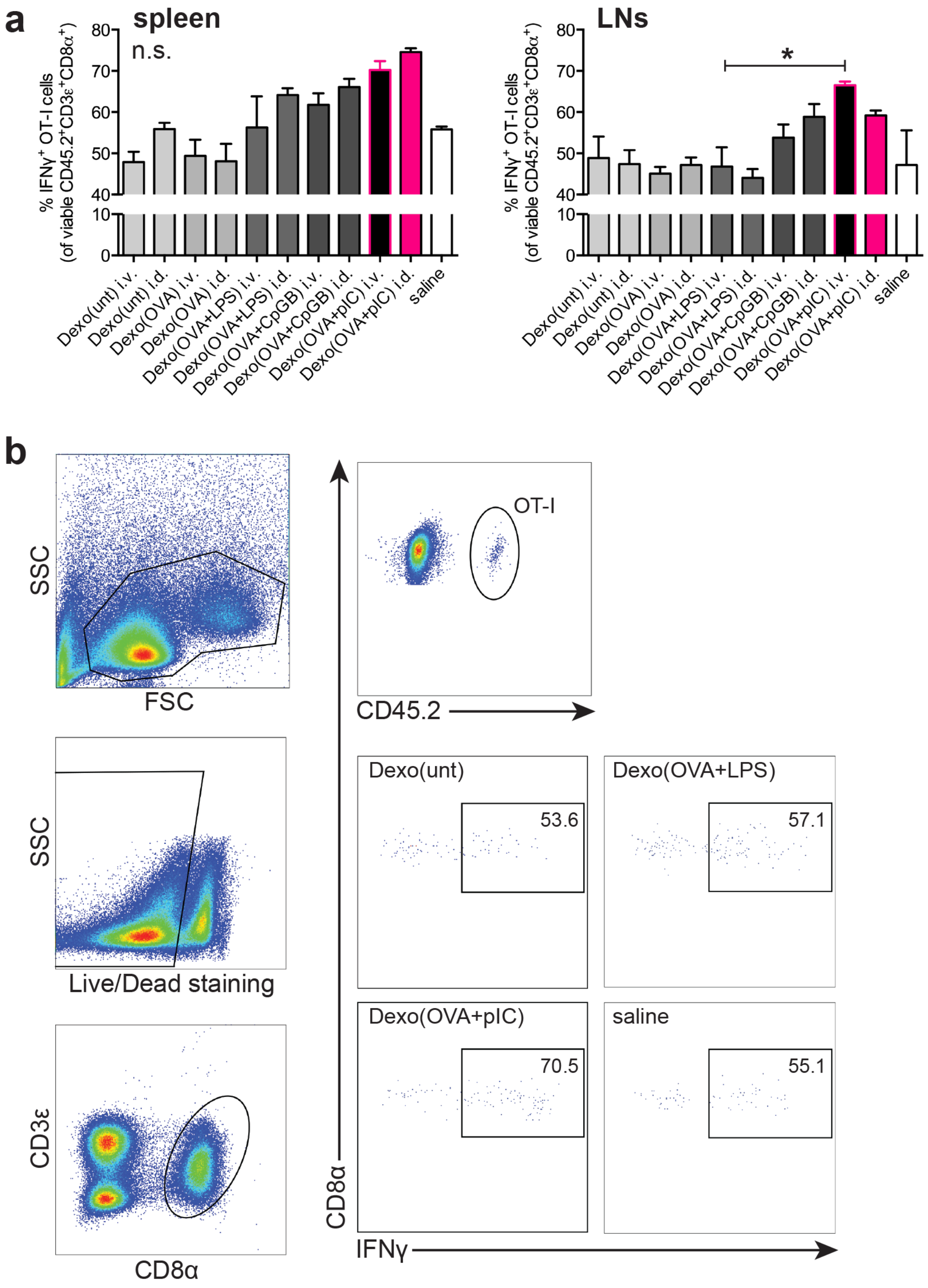

Supplementary figure-2

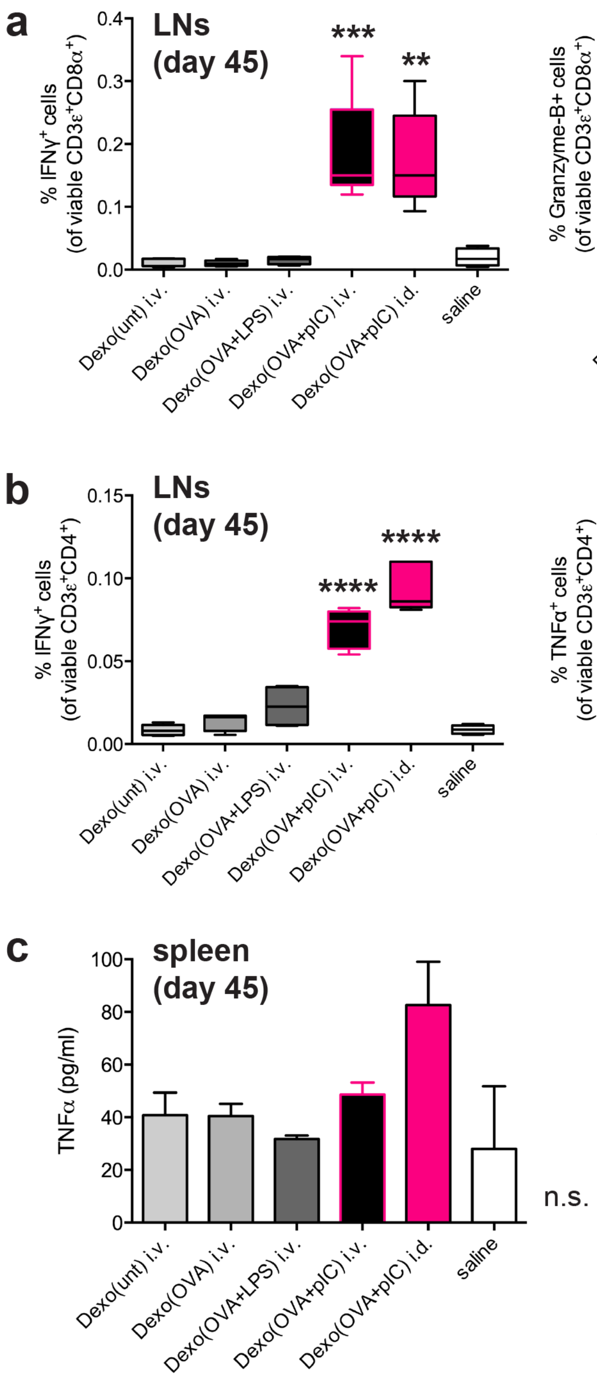

Supplementary figure-3

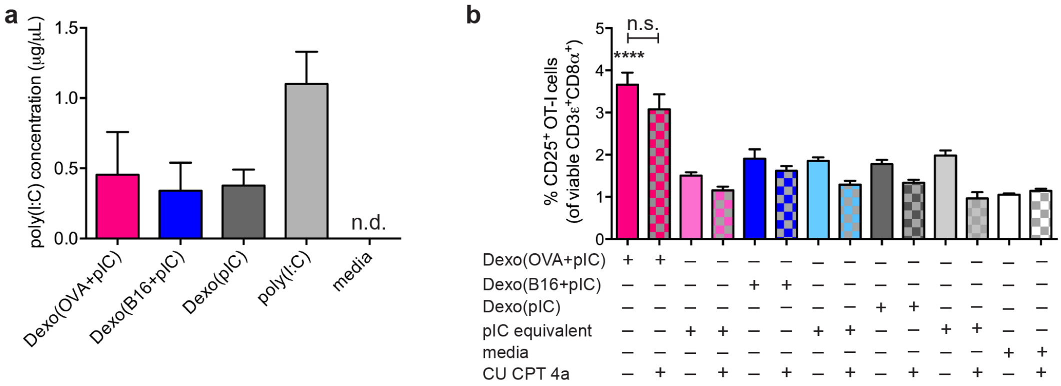

Supplementary figure-4

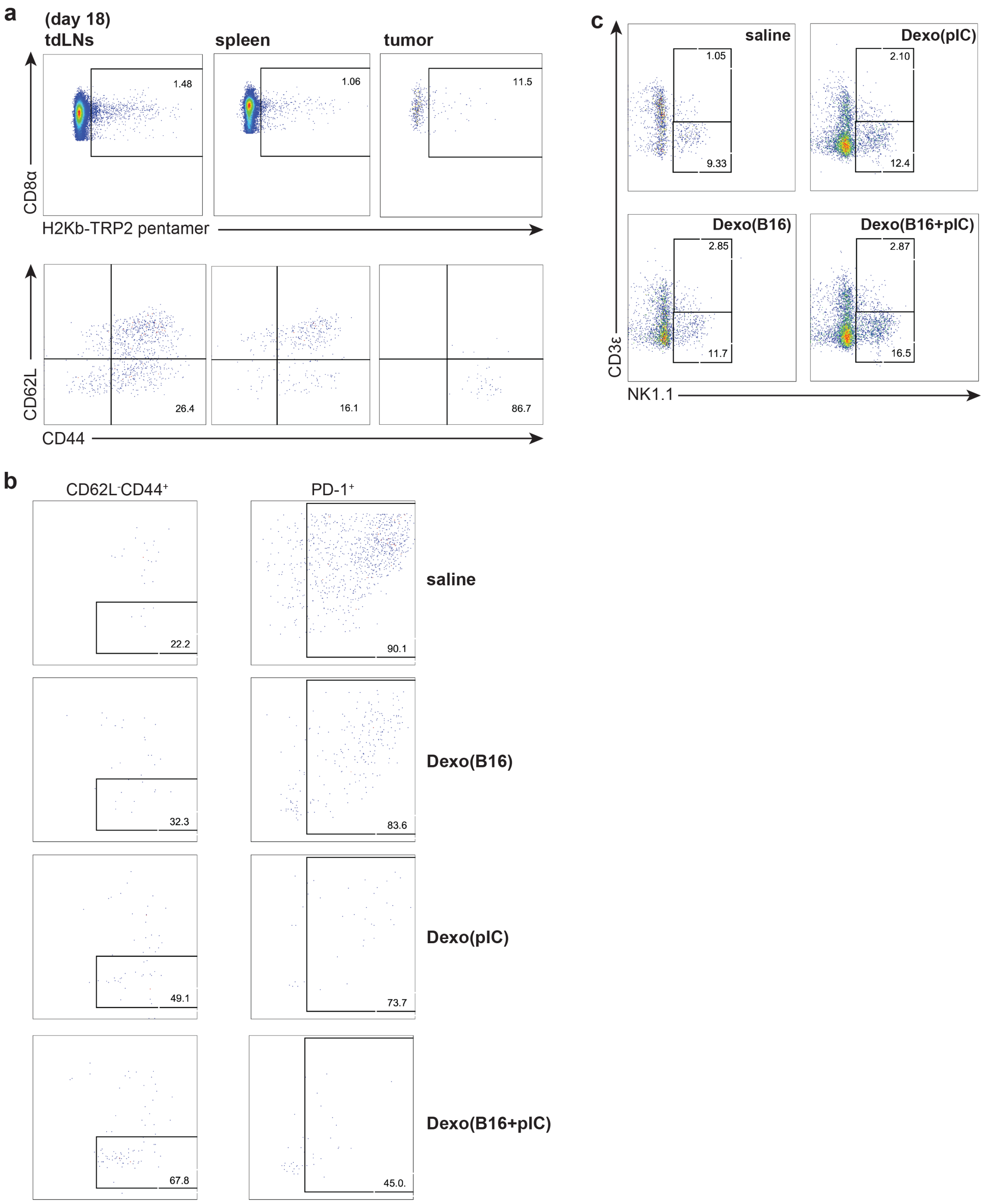

Supplementary figure legends

Supplementary figure 1. Vaccination with exosomes from OVA-loaded and poly(I:C)-activated DCs strongly activates proliferation and acquisition of effector functions of adoptively transferred OT-I OVA-specific CD8$^+$ T cells *in vivo*. (a) Effector functions of transferred CD45.2$^+$CD3ε$^+$CD8α$^+$ OT-I cells harvested from the spleen (left) or LNs (right) of mice vaccinated as indicated in Fig. 1a with the indicated Dexo formulation or saline were evaluated by detecting IFNγ expression by intracellular staining and flow cytometry. (b) Representative flow cytometric plots of LN cells stained for viability and CD3ε, CD8α, CD45.2 and IFNγ to identify adoptively transferred IFNγ$^+$ OT-I cells in mice vaccinated as in Fig. 1a with the indicated Dexo formulation administered i.d. or saline. Numbers represent the frequency of IFNγ$^+$ events in the population of viable CD45.2$^+$CD3ε$^+$CD8α$^+$ OT-I cells.
Data represent mean ± SEM from 2 independent experiments (N = 10). Statistical analysis was performed by one-way ANOVA and Bonferroni *post-hoc* test correction between the indicated experimental groups. * $P < 0.05$.

Supplementary figure 2. Vaccination with exosomes from OVA-loaded and poly(I:C)-activated DCs induces the expansion and acquisition of effector functions of endogenous OVA-specific CD4$^+$ and CD8$^+$ T cells with negligible OVA-specific antibody titers *in vivo*. (a) LN cells from mice vaccinated as in Fig. 3a with the indicated Dexo formulation or saline were collected on day 45 to measure acquisition of effector functions by SIINFEKL-specific CD8$^+$ T lymphocytes as detected by intracellular staining for IFNγ (left) and Granzyme-B (right) and flow cytometric analysis. (b) LN cells from mice vaccinated as in . 3a with the indicated Dexo formulation or saline were collected on day 45 to measure acquisition of effector functions by OVA-specific CD4$^+$ T lymphocytes as detected by intracellular staining for IFNγ (left) and TNFα (right) and flow cytometric analysis. (c) Splenocytes from vaccinated mice were collected on day 45 to measure TNFα secreted in the cell supernatant by ELISA.
Data represent mean ± SEM from 2 independent experiments (N = 10). Statistical analysis was performed by one-way ANOVA and Bonferroni *post-hoc* test correction. ** $P < 0.01$, *** $P < 0.001$, **** $P < 0.0001$ and n.s. = not significant for comparisons of Dexo(OVA+pIC) with Dexo(OVA+LPS).

Supplementary figure 3. Exosomes purified from poly(I:C)-activated DCs contain poly(I:C) carry-over but show greater antigen-specific immune stimulatory properties than equivalent doses of poly(I:C). (a) To detect and quantify poly(I:C) molecules in Dexo samples, murine TLR-3 reporter HEK-293T cells were treated with exosomes purified from poly(I:C)-activated DCs either loaded with OVA (Dexo(OVA+pIC)) or oxidized B16-F10

cells (Dexo(B16+pIC)) or no antigen (Dexo(pIC)). As a control to test the assay quantification, reporter cells were either treated with a known amount of poly(I:C) (1 μg/μL) or left untreated. (b) OVA-specific OT-I cells were co-cultured in the presence of bone-marrow-derived DCs and treated with 25 μg of the indicated Dexo formulation or with a dose of poly(I:C) equivalent to the amount of active poly(I:C) measured in the Dexo formulation as in (a). The TLR-3 specific inhibitor CU CPT 4a was also added in the indicated co-culture conditions to test the involvement of poly(I:C)-triggered TLR-3 activation in the stimulation of OT-I cells by Dexo. Activation of the OT-I cells was measured by flow cytometric analysis of viable $CD25^+CD3\varepsilon^+CD8\alpha^+$ OT-I cells.

Data represent mean ± SEM from two independent experiments (N = 6). Statistical analysis was performed by one-way ANOVA and Bonferroni *post-hoc* test correction. **** $P < 0.0001$.

Supplementary figure 4. Therapeutic vaccination with tumor cell antigen-loaded exosomes from poly(I:C)-activated DCs significantly reduces the growth of B16-F10 tumors and improves the survival of tumor-bearing mice by activating melanoma-specific $CD8^+$ T cells and promoting tumor infiltration of cytotoxic cells. (a) Representative flow cytometry dot plots of TRP-$2_{180–188}$ (SVYDFFVWL)-specific $CD8^+$ T lymphocytes (top) and of $CD62L^-CD44^+$ effector memory TRP2-specific $CD8^+$ T lymphocytes (bottom) harvested on day 18 from the tdLNs (left), spleen (middle) and tumor mass (right) of mice vaccinated as in Fig. 5a with Dexo(B16+pIC). Numbers indicate the frequency of gated cells among viable $CD3\varepsilon^+CD8\alpha^+$ cells (b) Representative flow cytometry dot plots of tumor-infiltrating $CD62L^-CD44^+$ effector memory (left) and exhausted PD-1^+ $CD8^+$ T lymphocytes (right) in mice treated as in Fig. 5a with the indicated Dexo formulation or saline. Numbers represent the frequency of gated events among viable $CD45^+CD3\varepsilon^+CD8\alpha^+$ cells. (c) Representative dot plots of tumor-infiltrating NK ($CD3\varepsilon^-NK1.1^+$) and NK-T ($CD3\varepsilon^+NK1.1^+$) cells from mice vaccinated as in Fig. 5a with the indicated Dexo formulation or saline. Numbers represent the frequency of gated events among viable $CD45^+$ cells.

Representative data from 2 independent experiments (N = 15).

Supplementary Methods

BCA, transmission electron microscopy, dynamic light scattering analysis and flow cytometry of Dexo

Total protein concentration of Dexo samples was measured by BCA assay (Thermo Fisher Scientific, Waltham, MA) following the manufacturer's instructions and it was used to determine the dose of Dexo for *in vitro* and *in vivo* studies. For transmission electron microscopy, 20 μg of Dexo were serially diluted in PBS and samples were transferred onto carbon-coated Formvar grids (SPI Supplies, Structure Probe Inc., West Chester, PA). Dexo

were then fixed in 1% glutaraldehyde, stained in uranyl-oxalate pH 7 and imaged with a Tecnai Spirit (FEI, Hillsboro, OR) transmission electron microscope. For dynamic light scattering analysis, exosomes samples were diluted in PBS and analysed with a Zetasizer instrument (Malvern, UK). For flow cytometric analysis, 25 μg of Dexo were adsorbed on magnetic beads coated with anti-CD81 Ab (Life Technologies). After washing, Dexo were stained with Abs specific for the surface markers CD63, H-2Kb (MHC-I), I-A/I-E (MHC-II), CD80 and CD86 in 2% FBS PBS solution. Samples were acquired on an LSR II cytometer (BD Biosciences) and data analyzed with FlowJo software (Tree Star).

Mass spectrometry (LC-MS/MS) and Western blot analysis

20 μg of Dexo were boiled at 70°C for 10 min in Laemmli buffer (Bio-Rad) and loaded onto a 12% polyacrylamide gel (Bio-Rad) for SDS-Page. For LC-MS/MS analysis, after electrophoresis gels were stained with SimplyBlue stain (Life Technologies) and the protein bands of interest were sliced and digested in trypsin solution prior to injection into LC-MS. Data analysis was performed using the Scaffold_4.4.4 software (Proteome Software, Portland, OR).

For western blot, after SDS-Page proteins were transferred onto an Immobilon PVDF membrane (Millipore) and the membrane was blocked in 5% skim milk PBS solution O/N at 4°C. Membranes were then incubated with primary Abs specific for Alix, CD81, OVA, TRP-2 or Tsg101 diluted in 5% skim milk PBS for 1 hr at RT, washed in 0.05% Tween-20 PBS and incubated with horseradish peroxidase-conjugated secondary Abs diluted in 5% skim milk PBS for 1 hr at RT. After extensive washing in 0.05% Tween-20 PBS, protein bands were visualized using SuperSignal West Pico Chemiluminescent substrate (Thermo Fisher Scientific) according to manufacturer's instructions.

For proteinase K digestion of Dexo samples, 20 μg of Dexo were incubated at 37°C in 50 μg/mL proteinase K (Qiagen, Limburg, The Netherlands) PBS solution for 10 min followed by 15 min at 95°C to deactivate the enzyme. Samples were then prepared as indicated above for SDS-Page and western blot.

Vaccination studies

For the OT-I adoptive transfer model of vaccination, on day 0, $CD45.2^+$ OT-I cells were purified from the spleen and LNs of OT-I transgenic mice by negative selection of $CD8\alpha^+$ T cells using the EasySep mouse CD8α T cell isolation kit (Stemcell Technologies, Grenoble, France) following the manufacturer's instructions. For CFSE labeling, OT-I cells were incubated in 1 μM CFSE (Life Technologies) PBS solution at 5×10^6 cells/mL at RT for 6 min and labeling reaction was quenched by adding an equal volume of 10% FBS PBS. $CD45.1^+$ C57BL/6-Ly5.1 recipient mice were anesthetized with isoflurane and 10^6 OT-I

cells/mouse were administered by tail vein injection. On day 1, recipient mice were anesthetized with isoflurane and vaccinated either intravenously by tail vein injection or intradermally into the 4 footpads with a dose of 50 µg of the indicated Dexo formulation. On day 6, recipient mice were euthanized to collect splenocytes and LN cells.

For the endogenous vaccination model, C57BL/6 mice were anesthetized with isoflurane and administered either intravenously into the tail vein or intradermally into the 4 footpads with 50 µg of the indicated Dexo formulation on day 0, 14 and 40. Blood was sampled on day 19 performing a small cut on the tail to collect peripheral lymphocytes and perform pentamer staining. On day 45, mice were euthanized to collect splenocytes, LN cells and blood serum.

Ex vivo restimulation, surface and intracellular staining and flow cytometry

Splenocytes were obtained by gently disrupting the spleen through a 70-µm cell strainer (Corning, NY), followed by extensive washing and red blood cell lysis. LNs were first digested for 45 min in RPMI 1640 GlutaMAX medium supplemented with 1 mg/mL Collagenase D (Roche, Basel, Switzerland).

Intracellular IFNγ of OT-I cells was determined after 2 hr of PMA (50 ng/mL)/ionomycin (1 µg/mL) treatment followed by 2 hr of BFA treatment (5 µg/mL) of the splenocytes and LN cells.

For T cell antigen-specific restimulation and intracellular cytokine staining, cells were cultured for 6 hr at 37 °C in the presence of 1 µg/mL SIINFEKL (GenScript, Piscataway, NJ) or 100 µg/mL OVA grade V protein for $CD8^+$ T cell or $CD4^+$ T cell restimulation, respectively, with the addition of 5 µg/mL BFA for the last 3 hr of culture.

Cells were then processed, counted and stained for viability and immunological markers to be analyzed by flow cytometry. Cells were first stained using Live/Dead fixable cell viability reagents (Life Technologies) according to the manufacturer's instructions and then stained with Abs specific for the surface markers CD45.2, CD3ε, CD4, CD8α, CD62L and CD44. Staining with PE-labeled H-2Kb/SIINFEKL pentamer was performed according to the manufacturer's instructions. For intracellular cytokine staining, cells were fixed in 2% paraformaldehyde solution, washed with 0.5% saponin (Sigma-Aldrich) in 2% FBS PBS solution, and incubated with Abs specific for IFNγ, Granzyme-B and TNFα diluted in saponin solution. After washing, cells were resuspended in 2% FBS PBS solution for analysis. Samples were acquired on an LSR II cytometer (BD Biosciences) and data analyzed with FlowJo software (Tree Star).

Alternatively, splenocytes were restimulated for 4 days in the presence of 100 µg/mL OVA grade V protein for the measurement of IFNγ, TNFα and IL-10 released in the supernatant by ELISA using Ready-SET-go! ELISA kits from eBioscience.

OVA-specific antibody ELISAs on serum samples were performed as described previously [36].

All cells were cultured in IMDM GlutaMAX medium supplemented with 10% FBS and 100 IU/mL penicillin-streptomycin.

Culture and oxidation of B16F10 cells

B16F10 (CRL-6475) cells were obtained from the American Type Culture Collection and cultured according to the instructions. Cells were checked for *Mycoplasma* prior to use; no additional authentication was performed. The procedure to obtain oxidation-dependent necrosis of tumor cells was previously published [31]. Briefly, B16-F10 cells were resuspended at 10^6 cells/mL in 60 μM HOCl solution, obtained by diluting the stock NaOCl in HBSS buffer, and incubated at 37°C for 1 hr. Cells were extensively washed in HBSS and either analyzed for viability using Live/Dead fixable cell viability reagent followed by flow cytometry or used for Dexo production or *ex vivo* restimulation.

B16F10 tumor vaccination studies

For restimulation with oxidized B16F10 cells and intracellular cytokine staining, splenocytes and tdLN cells were cultured for 6 hr at 37 °C in the presence of 10^5 HOCl-oxidized B16F10 cells, with the addition of 5 μg/mL BFA for the last 3 hr of culture.

Cells were then processed, counted and stained for viability and immunological markers to be analyzed by flow cytometry. Cells were first stained using Live/Dead fixable cell viability reagents and then stained with Abs specific for the surface markers CD45, CD3ε, CD8α, CD62L, CD44, PD-1 and NK1.1 Staining with PE-labeled H-2Kb/TRP-$2_{180\text{-}188}$ pentamer was performed according to the manufacturer's instructions. For intracellular staining of IFNγ and TNFα, cells were processed as indicated above. Samples were acquired on an LSR II cytometer and data analyzed with FlowJo software.

For imaging by confocal microscopy, tumors were fixed in 4% paraformaldehyde solution and frozen in OCT (Sakura, Osaka, Japan). 10 μm-thick sections were sliced and stained with primary Abs specific for CD45, CD3ε and PD-1 and fluorescently labeled secondary Abs (Life Technologies). Samples were mounted using ProLong Gold antifade reagent with DAPI (Life Technologies) and imaged with a LSM 700 inverted confocal microscope (Zeiss, Jena, Germany).

TLR-3 reporter assay and OT-I in vitro activation

Murine TLR-3 reporter HEK-293T cells were purchased from InvivoGen and cultured as indicated by the manufacturer's instructions. For the reporter assay, 2×10^4 cells were cultured with serial dilutions of poly(I:C) as standard curve or 10 μg of the indicated Dexo formulation for 24 hr at 37°C 5% CO_2. Engagement of TLR-3 resulting in NF-κB activation

was measured by QUANTI-Blue colorimetric assay (InvivoGen) as indicated by the manufacturer's instructions.

To measure *in vitro* activation of OT-I cells by Dexo, 10^5 OT-I cells were cultured in IMDM GlutaMAX medium supplemented with 10% FBS and 100 IU/mL penicillin-streptomycin together with 2×10^4 BMDCs in the presence of 25 μg of the indicated Dexo formulation or of a dose of free poly(I:C) equivalent to the amount of active poly(I:C) detected in the correspondent Dexo sample or left untreated for 24 hr at 37°C 5% CO_2. To block poly(I:C)/TLR-3 interactions, the TLR-3 specific inhibitor CU CPT 4a (Bio-Techne, Minneapolis, MN) was added to the culture media at 27 μM concentration, as previously published [45]. After incubation, cells were stained for viability using Live/Dead fixable cell viability reagents and with Abs specific for the surface markers CD3ε, CD8α and CD25. Samples were acquired on an LSR II cytometer and data analyzed with FlowJo software.
